# Supplementary figures and images for: CTRR-ncRNA: A Knowledgebase for Cancer Therapy Resistance and Recurrence Associated Non-coding RNAs
Source: Genomics Proteomics Bioinformatics. 2022 Oct 17;21(2):292–9. doi: 10.1016/j.gpb.2022.10.003 (PMC10626174; doi:10.1016/j.gpb.2022.10.003)

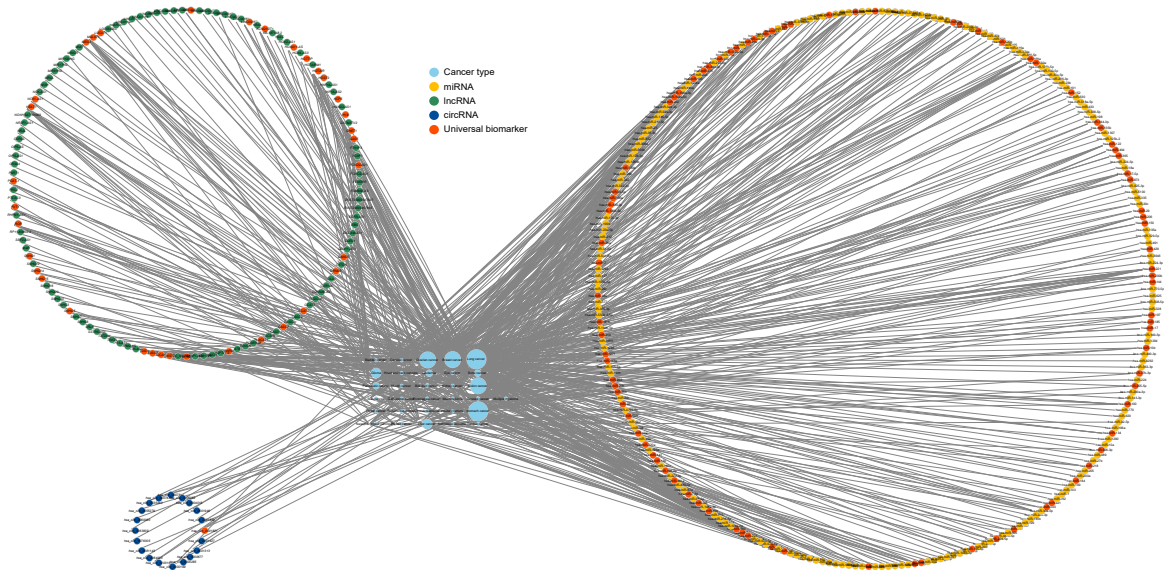

Supplement: Supplementary Figure S1 — An overview of the cancer–ncRNA network in CTR circRNA, circular RNA. [file mmc2.pdf]
